# Hypoxia differently modulates the release of mitochondrial and nuclear DNA

**DOI:** 10.1038/s41416-019-0716-y

**Published:** 2020-01-13

**Authors:** Amaelle Otandault, Jean-Daniel Abraham, Zahra Al Amir Dache, Abdelnaby Khalyfa, Isabelle Jariel-Encontre, Thierry Forné, Corinne Prévostel, Salem Chouaib, David Gozal, Alain R. Thierry

**Affiliations:** 1 IRCM, Inserm U1194, Institut de recherche en cancérologie de Montpellier, 208, avenue des Apothicaires, Montpellier, 34298 France; 2 Université de Montpellier, Montpellier, 34090 France; 3 Institut régional du cancer de Montpellier, Montpellier, 34298 France; 4 Department of Child Health and Child Health Research Institute, University of Missouri School of Medicine, Columbia, MO 65201 USA; 5 Institut de Génétique Moléculaire de Montpellier, University of Montpellier, CNRS, Montpellier, France; 6 INSERM UMR 1186, Integrative Tumor Immunology and Genetic Oncology, Gustave Roussy, EPHE, Fac. de médecine-Univ. Paris-Sud, University Paris-Saclay, Villejuif, 94805 France; 7 TRIPM, Gulf Medical University, Ajman, UAE

**Keywords:** Cancer metabolism, Diagnostic markers

## Abstract

**Background:**

We investigated the influence of hypoxia on the concentration of mitochondrial and nuclear cell-free DNA (McfDNA and NcfDNA, respectively).

**Method:**

By an ultra-sensitive quantitative PCR-based assay, McfDNA and NcfDNA were measured in the supernatants of different colorectal cell lines, and in the plasma of C57/Bl6 mice engrafted with TC1 tumour cells, in normoxic or hypoxic conditions.

**Results:**

Our data when setting cell culture conditions highlighted the higher stability of McfDNA as compared to NcfDNA and revealed that cancer cells released amounts of nuclear DNA equivalent to the mass of a chromosome over a 6-h duration of incubation. In cell model, hypoxia induced a great increase in NcfDNA and McfDNA concentrations within the first 24 h. After this period, cfDNA total concentrations remained stable in hypoxia consecutive to a decrease of nuclear DNA release, and noteworthy, to a complete inhibition of daily mitochondrial DNA release. In TC1-engrafted mice submitted to intermittent hypoxia, plasma NcfDNA levels are much higher than in mice bred in normoxia, unlike plasma McfDNA concentration that is not impacted by hypoxia.

**Conclusion:**

This study suggests that hypoxia negatively modulates nuclear and, particularly, mitochondrial DNA releases in long-term hypoxia, and revealed that the underlying mechanisms are differently regulated.

## Background

Hypoxia plays a key role in tumour progression, cell differentiation and metabolism, as well as in cell death and survival.^1–3^ The adaptation of cancer cells to the hypoxic tumour microenvironment is critical to their survival and proliferation.^4^ At the molecular level, cancer cells modify their metabolism, promote aerobic glycolysis and decrease mitochondrial oxidative phosphorylation, independent of cellular oxygenation levels.^5^ This process is known as the Warburg effect.^6–8^ Since that discovery, many studies demonstrated the impact of hypoxia on tumour progression, revealing a number of critical effects on angiogenesis,^9^ metastatic potential,^10^ protein synthesis,^11^ nuclear and mitochondrial DNA replication.^12,13^ Moreover, the aberrant angiogenesis and rapid cell proliferation create tumour areas with different degrees of hypoxia.^14^ Studies have shown that hypoxic tumour cells are more resistant to radiotherapy^15^ and to many commonly used chemotherapeutic agents.^16^ In clinical oncology, the presence of hypoxic cells in solid tumours is closely associated with a poor prognosis and survival in patients with many types of cancer.^17–19^ Epidemiological studies have associated higher mortality and increased cancer aggressivity in patients with intermittent hypoxia (IH) due to obstructive sleep apnoea.^20,21^ Moreover, recent studies of the plasma of mice under IH have shown a correlation between oxygen deprivation in the tumour microenvironment and the presence of circulating cell-free DNA (cfDNA).^22,23^ Interestingly, several reports have demonstrated the significant potential of cfDNA analysis in clinical oncology.^24^ For instance, cfDNA detection can allow the identification of minimal residual disease,^25,26^ the monitoring of drug efficacy or resistance,^27–29^ and prognosis of the disease.^30,31^ The potential of circulating cfDNA analysis was further shown in a recent analysis we performed on a large number of plasma samples taken from metastatic colorectal cancer (mCRC) patients, in which we compared the plasmatic concentrations of circulating cfDNA of nuclear (NcfDNA) and mitochondrial (McfDNA) origins. This analysis showed significantly higher NcfDNA concentrations in mCRC patients as compared to healthy subjects, while McfDNA concentrations were lower.^32–35^ However, our understanding of the structure and origins of cfDNA, of their pharmacokinetics and of the correlation between DNA release and tumour biology in different physiological states, especially hypoxia, remains limited.^36^ The aim of the present work is to study the effect of hypoxia on the release of DNA of both nuclear and mitochondrial origins. First, we set the appropriate cell culture conditions for optimising biological observations, in particular extracellular cfDNA stability and accumulation with incubation time. Thereafter, we determined the level and kinetics of the DNA release in normoxic and hypoxic conditions in cancer cell lines. We also used an in vivo autologous model to evaluate the amount of circulating NcfDNA and McfDNA in the plasma of mice engrafted with tumour cells, which were subjected to normoxia or IH as previously performed by our group.^22^

## Methods

### Cell culture and cell lines

Human colorectal cancer cell lines (SW620, SW480 and HCT116) were cultured in RPMI-1640 medium (Wako, Osaka, Japan). DiFi and DLD1 cell lines were cultured in Dulbecco’s modified Eagle’s medium (Life Technology, UK). All media were supplemented with 10% of foetal bovine serum (Eurobio) and 100 µg/mL of penicillin–streptomycin (Gibco™). For normoxic conditions, the cells were classically incubated at 37 °C in humidified atmospheric air with 5% CO_2_ addition. For hypoxic incubation, cells were placed in an incubator upon 0.5% O_2_, 94.5% N_2_ and 5% CO_2_. Cell lines were routinely tested for mycoplasma contaminations.

### Tumour animal models

C57BL/6J male mice (7 weeks old) were acquired from Jackson Laboratories (Bar Harbor, ME). Twelve mice were placed in designed environmental chambers and subjected to IH, in alternating cycles of 90 s (6% FIO_2_ (fraction of inspired oxygen in the air), followed by 21% FIO_2_, 20 cycles/h) for 12 h per day. A control group (*n* = 9) was exposed to continuous circulating room air (RA). After 2 weeks of exposure, 1.5 × 10^5^ TC1 cells (ATCC, Manassas, VA, USA) were suspended in 200 µL sterile phosphate-buffered saline and injected into the middle right flank of each animal that were then exposed to the same protocol (RA or IH) during 4 weeks. The tumour growth was monitored three times per week using a calibrated calliper. Carbon dioxide (CO_2_) gas was used for euthanasia, and the depth of anaesthesia was monitored by toe pinch, followed by cervical dislocation as per the institutionally approved ethical protocol. These procedures were always conducted between 9:00 and 11:00 a.m. in specially designated surgical room within the institutional vivarium. At the time of sacrifice (28 days after tumour injection), blood was collected from the tail, tumours were harvested for volume and weight measurements and processed for histologic and biochemical analyses. All experimental procedures were approved by The Institutional Animal Care and Use Committee of the University of Chicago (protocol# 2190). Plasma samples were sent to IRCM (France) for blind cfDNA measurements.

### Sample treatment and cfDNA extraction

All methods were performed according to the pre-analytical guidelines previously established by our group.^37^ Supernatants from cultured cancer cell lines or mice plasma were harvested in Eppendorf tubes, centrifuged to remove all cells (1200 × *g*; 10 min) and then frozen until use. After thawing, samples were centrifuged at 16,000 × *g* for 10 min at 4 °C to remove cellular debris and organelles.^38,39^ The supernatants were then transferred to 1.5 mL Eppendorf tubes and immediately extracted using the Qiagen Blood Mini Kit (Qiagen, CA), according to the manufacturer’s protocol. DNA was eluted from the column with 80 µL of elution buffer.

### Measurement of cfDNA concentration by qPCR and evaluation of MNR and DNA integrity

Table 1 describes the primers used to selectively amplify human and murine DNA sequences of nuclear and mitochondrial origins. The design of the primer system was performed under a stringent selective process. Quantifications of human NcfDNA and McfDNA were performed from the amplification of a 67 bp sequence on the *KRAS* gene^24,31^ and a 67 bp sequence on the MT-*COX-3* gene (short nuclear and mitochondrial amplicons, respectively).^32^ Fragments of 305 and 296 bp (long nuclear and mitochondrial amplicons, respectively) were amplified to evaluate DNA fragmentation by calculating the DNA integrity index (DII) (the ratio between long and short amplicon quantifications).^40,41^ Theoretically, the lower the DII, the more cfDNA is fragmented. The mitochondrial-to-nuclear ratio (MNR) corresponds to the ratio of the concentration of the McfDNA to that of NcfDNA, each calculated via short amplicon quantification. In order to quantify the murine circulating cfDNA, primers targeting murine *KRAS* (61 bp for short amplicon, 146 bp for long amplicon) or murine *COX-1* (114 bp for short amplicon) were used for NcfDNA and McfDNA quantifications, respectively. Quantitative PCR (qPCR) was performed in a final reaction volume of 25 μL, which was composed of 12.5 μL of PCR mixture (Bio-Rad iQ SYBR Green Supermix), 2.5 μL of each amplification primer (3 pmol/mL), 2.5 μL of PCR-quality water and 5 µL of DNA sample. Real-time qPCR was performed as follows: hot polymerisation activation-denaturation, performed for 3 min at 95 °C, followed by 40 repeated cycles at 95 °C for 10 s and then at 60 °C for 30 s. After amplification, melting curves were generated by increasing the temperature from 60 to 90 °C in increments of 0.2 °C, to confirm the specificity of the PCR product. Human McfDNA concentrations were calculated in ng/mL, using an internal standard curve composed of serial dilutions of a 3382 bp human plasmid vector containing a unique MT-CO3 gene sequence (ABM good®). The concentration and the purity of the vector solution were determined by measuring the optical density with a BioPhotometer® D30 spectrophotometer (Eppendorf). Human NcfDNA concentrations were calculated using an internal standard curve composed of serial dilutions of genomic DNA from human colorectal cancer cells previously quantified by a BioPhotometer® D30 spectrophotometer, and expressed as ng/mL. The murine genomic DNA from Promega was used to produce a standard curve. Concentrations of the standard curve were expressed as ng/mL and were used to determine the concentration of NcfDNA and McfDNA in the murine plasma. Mean values were calculated from triplicate reactions, and internal negative controls with PCR-quality water were routinely used.

**Table 1:** List and sequence of human and murine primers used for qPCR assays.

Human	Primer name	Sens	Sequence	TM (°C)	%GC	Amplicon size (bp)
Nuclear DNA	KRAS B2	Reverse	CCCTGACATACTCCCAAGGA	59.4		
	KRAS B1	Forward	CCTTGGGTTTCAAGTTATATG	54		67
	KRAS A1	Forward	GCCTGCTGAAAATGACTGA	54.5		305
Mitochondrial DNA	MT-COX3 F	Forward	GACCCACCAATCACATGC	56	55.6	
	MT-COX3 R 67	Reverse	TGAGAGGGCCCCTGTTAG	58.2	61.1	67
	MIT-COX3 R 296	Reverse	CTCAGAAAAATCCTGCGAAGA	55.9	42.9	296

Mouse	Primer name	Sens	Sequence	TM (°C)	%GC	Amplicon size (bp)
Nuclear DNA	MU KRAS 61–146 R	Reverse	GCTTCATTATCCTGCTTCC	56	47.4	
	MU KRAS 61 R	Forward	TTGATTCCTTGCTAGTTCT	52	36.8	61
	MU KRAS 146 F	Forward	TTCCCTGGGTTTTGGACTTA		45	146
Mitochondrial DNA	MU MT COX1 F	Forward	GTCCCACTAATAATCGGAGC	60	50	
	MU MT COX1 R REV C	Reverse	TGCTTCTACTATTGATGATGC	58	55.5	114

*KRAS* Kirsten rat sarcoma, *COX3* cytochrome *c* oxidase subunit 3, *COX1* cytochrome *c* oxidase subunit 1

### Stability of extracellular cfDNA and evaluation of daily released DNA

Supernatants from DLD1 and SW620 cells, previously cultured during 24 h in T25 flasks (1 million cells in 10 mL culture medium) were harvested after a 24-h period of culture, centrifuged and then placed in an incubator at 37 °C for 4 days, to evaluate the intrinsic stability of extracellular DNA over time independently of the cells. Each day, DNA was extracted from 400 µL of supernatant and McfDNA and NcfDNA concentrations were evaluated by qPCR as described previously. Short and long amplicons were quantified to measure concentrations of both NcfDNA (67 and 305 bp, respectively) and McfDNA (67 and 296 bp, respectively). This allowed us to establish the equation necessary to estimate the amount of daily released extracellular DNA every 24 h in the culture medium:

$$\left[ {{\mathrm{cfDNA}}} \right]_{{\mathrm{neo}}{\mbox{-}}{\mathrm{released}}} = \left[ {{\mathrm{cfDNA}}} \right]_{{\mathrm{measured}}} - \left[ {{\mathrm{cfDNA}}} \right]_{{\mathrm{stable}}}.$$

### Statistical analysis

Data are expressed as the mean (±) standard deviation. A non-parametric statistical Mann–Whitney *U* test was used to compare different groups of mice (GraphPad Prism software V7). Correlations were assessed using Spearman’s non-parametric method. In all figures, statistical analyses were performed at the conventional significant *p* value: *p ≤ 0.05, **p ≤ 0.01, ***p ≤ 0.001 and ****p ≤ 0.0001.

## Results

### NcfDNA and McfDNA stability in supernatants from DLD1 and SW620 cell cultures

**Fig. 1:**
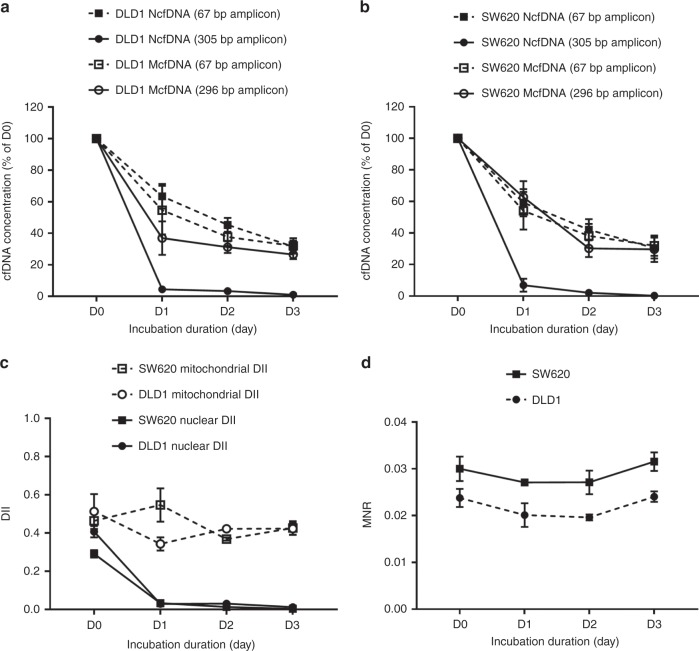
cfDNA stability in cell culture media. After a 24-h incubation period of the DLD1 and SW620 cell lines (one million cells in T25 flask), the medium was removed, centrifuged and incubated at 37 °C in 6-well plates. The concentration of cfDNA was monitored every day (D0, D1, D2 and D3) by qPCR, targeting *KRAS* or *COX3* genes and quantifying amplicons of 67 and 305 bp from nuclear cfDNA, and quantifying amplicons of 67 and 296 bp from mitochondrial cfDNA. The evaluation of cfDNA concentrations (in percentage of D0) is shown according to the cell line (**a**, **b**); **c** mitochondrial and nuclear DII; **d** MNR. Average values with (±) standard deviations are represented; results having been reproduced in three independent experiments, each performed in triplicate. cfDNA: extracellular cell-free DNA; DII: DNA integrity index; MNR: mitochondrial-to-nuclear DNA ratio; *KRAS*: Kirsten rat sarcoma; *COX3:* cytochrome *c* oxidase subunit 3.

As shown in Fig. 1a (DLD1 cell line) and Fig. 1b (SW620 cell line), the concentration of NcfDNA, measured via the detection of the long nuclear amplicon (black circles, full lines), dropped rapidly at D1: only 4% (DLD1 cells, Fig. 1a) and 7% (SW620 cells, Fig. 1b) of the concentration measured at D0 could be recovered. On the other hand, NcfDNA, measured via the detection of the short amplicon (black squares, dashed lines), remained relatively stable from D1 to D3 after a loss of 40% between D0 and D1, in DLD1 (Fig. 1a) or SW620 (Fig. 1b) supernatants. Likewise, McfDNA concentrations (67 and 296 bp amplicons) decreased between D0 and D1 (40% loss), but then remained stable after D1. This stability was highlighted by the mitochondrial DII value, which remained constant at 0.5 (Fig. 1c, dashed lines), in both cell lines. These profiles should be compared to the significant decrease of nuclear DII from 0.4 at D0 to 0.01 at D3 (Fig. 1c, full lines). Noteworthy, MNR values, calculated from short amplicons measurements, remained constant throughout the study delay, whatever the cell line (Fig. 1d). Thus, we show the high stability of short extracellular DNA amplicons of nuclear and mitochondrial origins in cell culture medium in the absence of cells: 60% of NcfDNA and McfDNA were recovered after 24 h of incubation (as compared to D0 of incubation), and 40% of this amount is recovered after 48 h of incubation. These results are of importance and will be used to calculate the daily release of DNA in normoxic vs. hypoxic conditions.

### Release of DNA in the supernatant of colorectal cancer cell lines

**Fig. 2:**
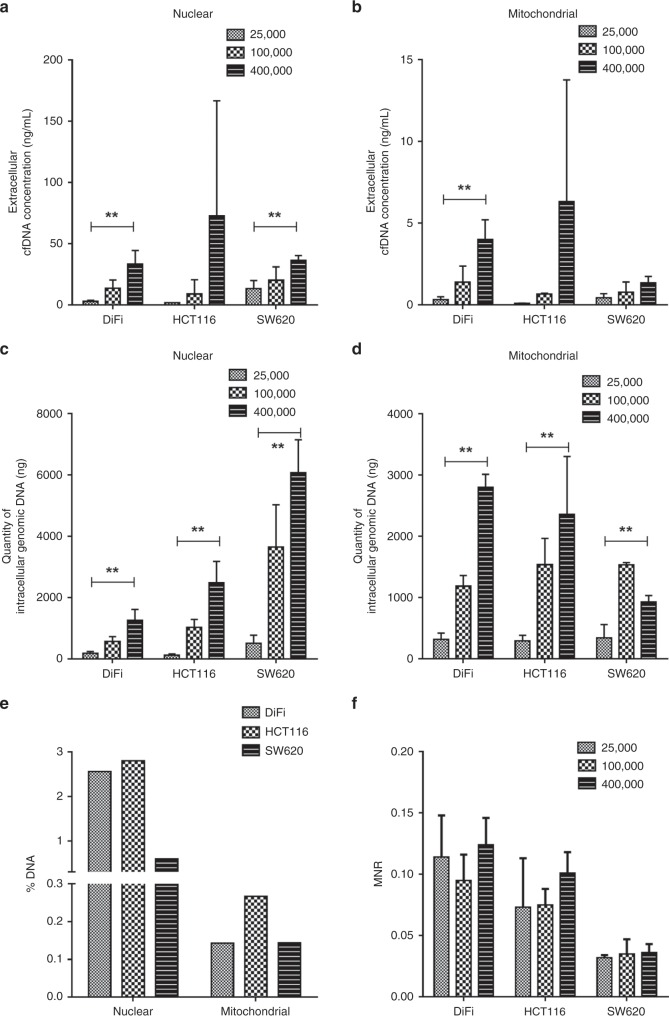
Release of cfDNA by colorectal cancer cells in culture. Culture media and cell pellets from colorectal cell lines (DiFi, HCT116, SW620) were harvested after 6 h of incubation at a different number of cells per well (25,000, 100,000 or 400,000). Extracellular and intracellular DNA of nuclear (**a**, **c**, respectively) or mitochondrial origin (**b**, **d**, respectively) were measured by qPCR, as described in the Methods section, and estimation was made of the percentage of DNA released in culture medium as compared to intracellular DNA (**e**) and of MNR (**f**). Average values with (±) standard deviations are represented, results having been reproduced in four independent experiments, each performed in triplicate. MNR: Mitochondrial-to-nuclear DNA ratio. *P* values significance: **P < 0.01.

In order to address the question of the impact of the cell type on DNA release, different colorectal cancer cell lines (DiFi, HCT116 or SW620) were seeded at different concentrations (25,000, 100,000 or 400,000 cells per well). After 6 h of incubation, culture media and cells were collected and processed in order to quantify NcfDNA and McfDNA by qPCR. As shown in Fig. 2, levels of NcfDNA (Fig.2a) and McfDNA (Fig. 2b) increased with the number of starting cells, regardless of the cell line tested. The same observation can be made regarding intracellular DNA (evaluated with the same qPCR protocol as the supernatants), which showed increased amounts of genomic DNA (Fig. 2c) and mitochondrial DNA (Fig. 2d) in cell pellets. Noteworthy, we also observed that the proportion of cfDNA as compared to intracellular DNA is fairly constant, whatever the cell line: between 0.6 and 2.8% of nuclear DNA was released from cells in 6 h (Fig. 2e). Lower proportions of mitochondrial DNA (from 0.14 to 0.22% of total mitochondrial DNA) were found in the supernatants, as compared to nuclear cfDNA. Finally, we were able to confirm the remarkable stability of the MNR parameter in cell supernatants, regardless of the number of cells (Fig. 2f). It is also interesting to note that the MNR value varied according to cell type: due to a lower mitochondrial DNA release, the SW620 cell line showed relatively lower MNR values in the extracellular medium, compared with the DiFi and HCT116 cell lines.

### Hypoxia effect on the release of DNA in SW620 cancer cell lines

**Fig. 3:**
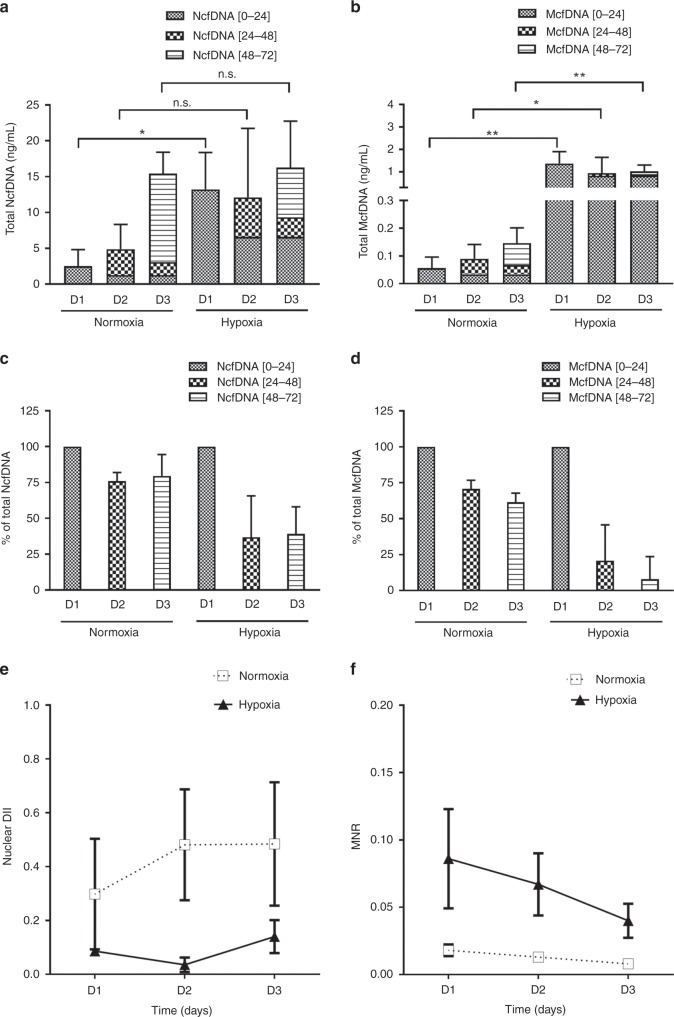
cfDNA release upon normoxic and hypoxic conditions. After seeding SW620 cells for 24 h, the culture medium was replaced with a fresh medium and cells were placed in two different incubators: one with normoxic conditions (21% O_2_) and the other with hypoxic conditions (0.5% O_2_). Different aliquots of the same supernatant were sampled during 3 days (D1–D3, days of sample collection). Total concentrations of nuclear (**a**) and mitochondrial (**b**) extracellular cfDNA were quantified by qPCR. cfDNA [0–24] corresponds to the concentration of released DNA during the first 24 h; cfDNA [24–48] corresponds to the concentration of released DNA between the 24th and 48th hour of incubation; cfDNA [48–72] corresponds to the concentration of released DNA between the 48th and 72nd hour of incubation. The proportions of nuclear or mitochondrial DNA newly released during each period, as compared to the total concentration measured each day, are shown in **c**, **d**, respectively; **e** shows the nuclear DII of supernatants from cells cultured in normoxia (white square, dashed line) or in hypoxia (dark triangle, solid line); **f** shows the MNR of supernatants from cells cultured in normoxia (white square, dashed line) or in hypoxia (dark triangle, solid line). Values are presented as mean (±) standard error of four independent wells, each performed in triplicate. cfDNA: extracellular cell-free DNA; DII: DNA integrity index; MNR: mitochondrial-to-nuclear DNA ratio; n.s.: not significant. *p*-Values significance: *p < 0.05, **p < 0.01, ***p ≤ 0.001 and ****p ≤ 0.0001.

After seeding SW620 cells for 24 h, the culture medium was replaced with a fresh medium and cells were cultured either in normoxic conditions or hypoxic conditions. Sequential aliquots of the same supernatant were sampled for 3 days (D1–D3). In normoxic condition, the total concentrations of NcfDNA and McfDNA increased during the 3 days of incubation, from 2.5 to 15.4 ng/mL (*p* = 0.0002) and from 0.05 to 0.14 ng/mL (*p* = 0.0194) respectively (Fig. 3a, b, normoxia panels). Under hypoxic conditions, total NcfDNA concentration was significantly higher in the first day of hypoxia (five times greater than under normoxic conditions; *p* = 0.01), and stabilised over time at 16.2 ng/mL at D3 (Fig. 3a, hypoxia panel). The total McfDNA concentration increased significantly in the first day of hypoxia (25 times greater than under normoxia; *p* = 0.004) and decreased slightly throughout the period of the study (1.37–0.77 ng/mL; Fig. 3b, *p* = 0.0436). When examining DNA release within one day, different cell behaviours can be distinguished. Indeed, owing to the results described in Fig. 1, we know that the concentrations of cfDNA measured at D2 include 60% of DNA released during the [0–24 h] period, while cfDNA measured at D3 include 60% of DNA released during the [24–48 h] period and 40% of DNA released during the [0–24 h] period. Thus, the daily release of DNA can be calculated on the basis of the following algorithm:

$${\mathrm{cfDNA}}_{{\mathrm{[0 - 24h]}}} = {\mathrm{cfDNA}}_{{\mathrm{D1}}},$$

$${\mathrm{cfDNA}}_{{\mathrm{[24 - 48h]}}} = {\mathrm{cfDNA}}_{{\mathrm{D2}}} - {\mathrm{60\% }}\,{\mathrm{cfDNA}}_{{\mathrm{[0 - 24h]}}},$$

$${\mathrm{cfDNA}}_{{\mathrm{[48 - 72h]}}} = {\mathrm{cfDNA}}_{{\mathrm{D3}}} - {\mathrm{60\% }}\,{\mathrm{cfDNA}}_{{\mathrm{[24 - 48h]}}} - {\mathrm{40\% }}\,{\mathrm{cfDNA}}_{{\mathrm{[0 - 24h]}}}.$$

Consequently, under normoxic conditions, daily nuclear and mitochondrial DNA releases account, respectively, for 70 and 65% of the total concentrations measured at D2, and for 80 and 62% of the total concentrations measured at D3 (Fig. 3c, d, normoxia panels). Under hypoxia, daily release of nuclear DNA decreased down to 50% of the total NcfDNA concentration at D3, while daily mitochondrial DNA release dropped below 0.1% of the initial concentration by D3 (Fig. 3c, d, hypoxia panels). This difference of profile between normoxia and hypoxia is also illustrated in Fig. 3e, f. The nuclear DII remained low under hypoxia during the 3 days of the study (0.03–0.14; black triangles; Fig. 3e), as compared to normoxia (0.299–0.48; white squares; Fig. 3e). MNR also progressively decreased from 0.09 down to 0.04 in hypoxia, while remaining very low in normoxia (from 0.008 to 0.018; Fig. 3f).

### Hypoxia influences tumour weight and plasma cfDNA levels in grafted mice

**Fig. 4:**
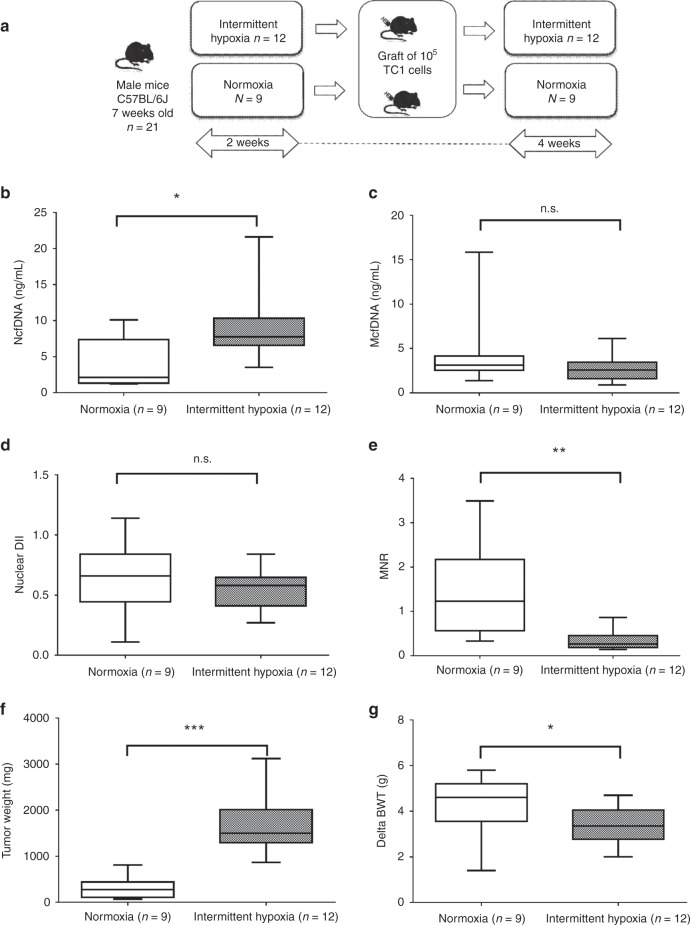
In vivo effects of intermittent hypoxia. **a** Flow chart of the in vivo experiment: C57BL/6j mice were pre-exposed during 2 weeks to either room air or intermittent hypoxia, and then injected with 10^5^ TC1 cells. Blood collection was performed 4 weeks after the graft. The concentrations of circulating cfDNA in plasma from mice subjected to normoxia (open boxes) or hypoxia (grey boxes) were evaluated by qPCR by targeting murine *KRAS* (**b**) or *COX1* (**c**) genes. This allowed the evaluation of the nuclear DII (**d**) and the MNR (**e**). The effect of hypoxia on tumour weight (**f**) and mouse body weight gain (**g**) are also presented. The line from end to end of the whisker represents the minimum and maximum values of the data, the line within each box represents the median and the lower and upper boundaries of the box indicate the first and third quartiles, respectively. Significant differences between normoxia and intermittent hypoxia were estimated using the Mann–Whitney test. *P* values significance: *p < 0.05, **p < 0.005 and ***p < 0.0005. NcfDNA: nuclear cell-free DNA; McfDNA: mitochondrial cell-free DNA; DII: DNA integrity index; MNR: mitochondrial-to-nuclear DNA ratio; dBWT: delta body weight; n.s.: not significant.

Twenty-one C57BL/6j mice engrafted with murine TC1 cells were used for in vivo experiment; nine of these mice were exposed to normoxia, while twelve were exposed to IH according to the experimental procedure depicted on Fig. 4a. Plasmatic circulating cfDNA concentrations were assessed in both conditions. Data showed a significant increase of the NcfDNA concentration in plasma from mice under IH as compared to normoxia (Fig. 4b, *p* = 0.0142). Conversely, McfDNA concentration was slightly decreased (but not significantly; Fig. 4c, *p* = 0.1886). As a result, IH greatly reduced the MNR in the plasma of grafted mice (Fig. 4e, *p* = 0.0022). In contrast, no statistical difference was observed for nuclear DII (Fig. 4d, *p* = 0.2861).

As shown in Fig. 4f, IH increased tumour weight (*p* = 0.0001). Conversely, its impact on mice growth (delta body weight) was slightly negative (Fig. 4g; *p* = 0.0359). Correlations between tumour weight and other parameters were assessed in the grafted mice: NcfDNA is positively correlated to tumour weight (Supplementary Fig. 1a; *r* = 0.5992; *p* = 0.0041), whereas MNR is negatively correlated to tumour weight (Supplementary Fig. 1d; *r* = −0.5054; *p* = 0.0194). McfDNA and nuclear DII are not significantly correlated to tumour weight (Supplementary Fig. 1b, c, respectively).

## Discussion

DNA molecules of nuclear and mitochondrial origins are found in the extracellular compartment, in vitro in the media of cell culture^42,43^ and in vivo in the physiological fluids.^44,45^ While the analysis of circulating DNA from plasma is now optimised and standardised,^37^ only poor experimental works have been performed with using cell culture. To accurately observe the effect of hypoxia on cells, we first carried out a study on standardising cell culture conditions to avoid any bias. We first examined the stability over time of NcfDNA concentration, as detected via the amplification of a short DNA sequence compared to a long DNA sequence to further estimate the DII as it was performed in vivo.^41^ The biphasic shape of the NcfDNA concentration curves suggests that at least two structural entities of different stability coexist in the pool of fragments over 67 bp. In contrast, fragments over 305 bp highly decrease with time of incubation down to nearly disappear after a few days of incubation. Thus, we can conclude that fragments of NcfDNA over 305 bp appear less stable than those higher than 67 bp in these in vitro conditions (almost 40% being still present following 2 days incubation in cell culture supernatant). We previously showed that targeting a sequence as short as possible enables to improve and consequently to accurately determine cfDNA concentration. The decrease of nuclear DII observed in our stability study, corresponding to the degradation of NcfDNA into fragments between 67 and 305 bp, can be explained by the presence of nucleosomal structures or transcription factor-associated complexes, which constitute 90–98% of the fragments detected in CRC patients.^46^ Conversely, McfDNA concentration is less impacted by incubation time, regardless of the targeted sequence length. This result indicates structural differences between nuclear and mitochondrial DNA in cell culture supernatant. McfDNA appears more stable as compared to NcfDNA, certainly due to different structural complexes, not yet characterised. Our group has recently demonstrated that cell culture medium could contain cell-free circulating respiratory-competent mitochondria or cell membrane- or debris-associated mitochondria,^47^ which could be one of the reasons why McfDNA seems protected. Noteworthy, the ratio between McfDNA and NcfDNA concentrations, which corresponds to the MNR, is highly stable and reproducible in cell-free culture medium. This observation is confirmed in cell model, as MNR, although varying according to the cell line, is constant regardless of the number of cells. This result indicates that MNR could be considered as a relevant biomarker, as it appears less influenced by collateral factors such as DNA conservation or cell number.^34^

In cancer cell models, our data revealed a rapid and significant release of DNA into the culture medium. A rapid release of nuclear DNA was also reported by Wang et al.^48^ in T47-D (80 ng/mL) and MDA-MB-231 (12 ng/mL) breast cancer cells over a short period of time (6 h of incubation). In addition to this, release of nuclear DNA was also observed during the first 24 h incubation in the culture medium of the human 143 B osteosarcoma cell line.^49^ As expected, we observed in our study that NcfDNA and McfDNA concentrations increased with the number of cells, corresponding to about 2% and 0.1% of the intracellular content, respectively, whatever the cancer cell lines. This difference of proportion may be explained by different mechanisms implied in either nuclear or mitochondrial DNA release. It should be noted that this 2% of NcfDNA is approximately equivalent to the extracellular release of one chromosome in 6 h, highlighting the high genomic dynamics as well as the importance of DNA release in extracellular space, which cannot therefore be considered as a marginal mechanism. From a physiological point of view, these data are of great importance because extracellular nucleic acids have been described as biologically active in inflammatory processes, and as potentially active in cell transformation and communication.^50^

The study of the DNA release and stability allowed us to provide an in vitro experimental model avoiding bias as much as possible in the following observations. Indeed, we took into consideration the respective stability of NcfDNA and McfDNA and the accumulation bias by using an algorithm allowing to delineate the daily rate of DNA release. Hence, our data revealed that hypoxia has a critical impact on cfDNA concentration in the supernatant of SW620 human colorectal cancer cell line, as compared to normoxic conditions, within the first 24 h of incubation. After this period of time, cfDNA concentrations did not increase further upon hypoxia. Inversely, cfDNA concentrations increase with normoxia, which allows a progressive accumulation of cfDNA in the cell supernatant. Furthermore, thanks to our stability study and the use of our algorithm, we reveal for the first time the different kinetics of nuclear and mitochondrial DNA release, revealing a biphasic behaviour of the cells submitted to hypoxia: a high release during the first 24 h followed by a reduced release at days 2 and 3. This effect was much more pronounced for mitochondrial DNA, the release of which is completely abrogated at day 3. This difference clearly indicates that the mechanisms implied in nuclear and mitochondrial DNA releases are different from each other.

The main mechanisms of nuclear DNA release described in the literature are cell death (apoptosis and necrosis) and active secretion, in particular through microparticle release.^36,51^ Several structures such as exosomes, apoptotic bodies, nucleosomes and virtosomes, which may contain extracellular DNA and which have been shown to be positively regulated by hypoxia, have been described.^51–53^ Moreover, other studies have shown that increased autophagy in response to hypoxic stress allows cell survival or death.^54,55^ In addition, under hypoxic conditions, the endogenous antioxidant response of cells may be insufficient, resulting in an intracellular increase in reactive oxygen species (ROS),^56^ and the increased release of nuclear and mitochondrial DNA. It may thus be speculated that DNA release may be increased by hypoxia via exosome production, ROS or autophagy in the early steps of our study. However, when the amount of ROS of a cell is too high, the necrotic activity of p53 could be induced, therefore promoting cell death.^57,58^ Indeed, regulated necrosis includes multiple cell death modalities, resulting in the loss of mitochondrial inner membrane potential, disruption of ATP production, mitochondrial dysfunction and consequent necrosis. This could explain our observation that mitochondrial DNA release is more affected than nuclear DNA by late incubation upon hypoxic conditions.^59^ Furthermore, it was shown that mitochondria undergo fission and fusion continually in response to changes in the extracellular environment: hypoxia promotes the production of ROS that cause an increase in mitochondrial fission.^60^ Noteworthy, recent studies highlighted modifications in the expression of genes involved in oxidative phosphorylation,^61^ linking hypoxia to mitochondrial adaption to this anaerobic environment. Under hypoxia, glucose consumption increases to maintain ATP production using less efficient anaerobic glycolysis, which induces major structural and dynamical changes characterised by impairment of fusion process that leads to mitochondrial depolarisation, and finally loss of mitochondrial DNA.^62,63^

Under normoxic conditions, the MNR remains stable over time. This suggests a balance between cell growth and the release of nuclear DNA and mitochondrial DNA. Conversely, hypoxia leads to an imbalance in this stability. These results suggest that the regulations of mitochondrial and nuclear releases could be valuable parameters to follow-up hypoxia in patients. To date, multiple approaches exist to detect hypoxia either directly or indirectly.^64^ Tumour hypoxia was shown to be a prognostic biomarker, as it was shown that extracellular vesicle-associated genes could correlate with hypoxic microenvironment and predict recurrence in lung adenocarcinoma.^65^ Evaluating hypoxia could also be indicative for the selection of patients who would most benefit of chemo-radiotherapy.^66^

The in vivo effect of hypoxia on the concentration of circulating NcfDNA and McfDNA was evaluated in the plasma of mice engrafted with an autologous lung epithelial tumour model (TC1) cell line. As would be expected from our in vitro results, plasmatic NcfDNA concentration is highly correlated to tumour weight and size, both of which are increased in mice bred in a hypoxic atmosphere, as has been observed in the previous study.^23^ Noteworthy, circulating McfDNA is weakly related to hypoxia in the mice model, as its plasmatic concentration is not significantly different in both groups of mice. As a result, McfDNA is not correlated to tumour weight. This result is in phase with the in vitro model, since we found that after a rapid and huge increase of its concentration in extracellular medium, mitochondrial DNA release is then negatively regulated after 24 h of incubation, and to a higher extent than nuclear DNA. The lack of difference in McfDNA levels in murine plasma between hypoxia and normoxia may also be explained by the fact that, due to structural similarities with bacterial DNA, mitochondrial components, including mitochondrial DNA itself, are particularly likely to activate the immune system, leading to their degradation.^67^ This hypothesis should be tested by the use of immunosuppressed xenografted mice. Nevertheless, the McfDNA levels can be used to establish the MNR, which permits a more accurate discrimination between normoxic and hypoxic mice than could be done if using NcfDNA levels alone. It would therefore appear that McfDNA measurement allows a normalisation of individual differences, highlighting the discriminative power of NcfDNA.

The results of our study must be interpreted within the context of their limitations and strengths. Despite colorectal cancer cell lines appear to cover the genetic, transcriptional and phenotypic constitution of cancer cells, their characteristics may be far from those of in vivo tumours, as most in vitro cell lines are derived from rapidly growing, aggressive transplantable tumour lines. Furthermore, carcinoma cells in in vivo models form 3D structures that interact with the surrounding extracellular matrix and cells of the tumour stroma. These interactions have been shown to be essential for tumour development and progression, which prominently alter signalling pathways in both the tumour cells and the stromal cells.^68,69^ These preliminary results will need to be consolidated by analysis on a larger number of cell lines and mice. To obtain a more complete picture of these processes, it will be necessary to identify the release mechanisms in order to establish how and why, in hypoxic conditions, the high release of mitochondrial DNA is transient and subsequently repressed, while that of nuclear DNA is much less affected.

In summary, this is the first study to delineate concomitantly the effect of hypoxia on the levels of nuclear and mitochondrial cfDNA in cell and murine models. We have clearly shown that cancer cell lines release high amounts of mitochondrial and nuclear DNA in the extracellular compartment, and that hypoxia strongly influences the amount and manner in which these are released. We have also demonstrated that cells are able to regulate differentially the daily rate of release of DNA according to hypoxic conditions. In the grafted mice model, we have shown that MNR, which is correlated to tumour size and weight, may characterise the hypoxic condition of the tumour. While it does not fully recapitulate mCRC in patients, our in vivo study using mice engrafted with TC1 cells does suggest that analysis of MNR could be relevant in evaluating the degree of hypoxia of human tumours. Moreover, the amount of NcfDNA in plasma could be indicative of tumour size. Our study is a preliminary one, but could pave the way for the development of blood tests, which would evaluate cancer progression or residual disease.

## Supplementary information

191211_supplementary_figure1

## Data Availability

Data are available upon request to the corresponding author, Dr. A.R. Thierry.
