# Supplementary figures and images for: Hypoxia differently modulates the release of mitochondrial and nuclear DNA
Source: Br J Cancer. 2020 Jan 13;122(5):715–25. doi: 10.1038/s41416-019-0716-y (PMC7054557; doi:10.1038/s41416-019-0716-y)

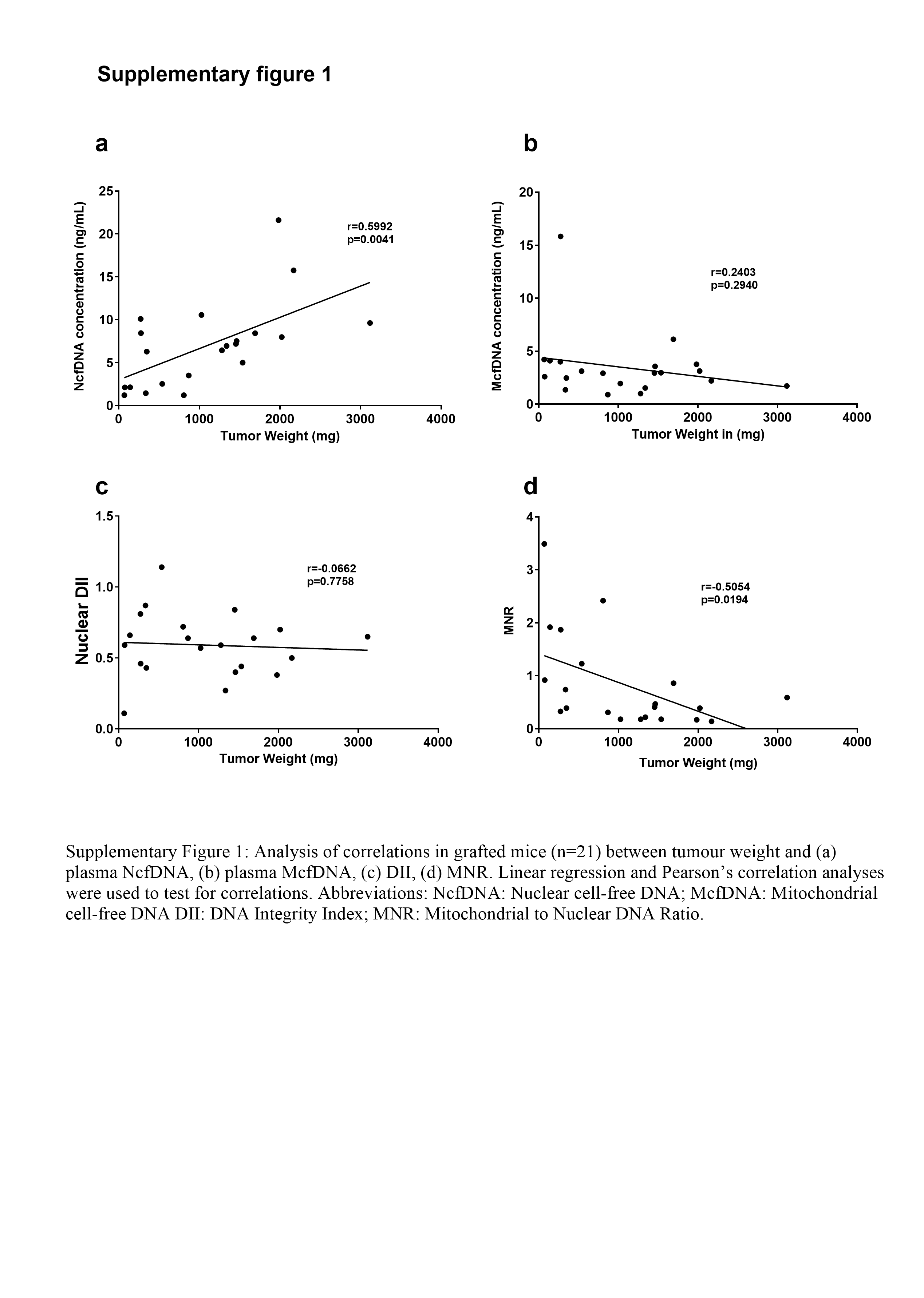

Supplement: Supplementary file 1 — 191211_supplementary_figure1 [file 41416_2019_716_MOESM1_ESM.tif]
